# Effects of pretreating wheat middlings and sunflower meal with fiber degrading enzymes on components solubilization and utilization in broiler chickens

**DOI:** 10.1093/tas/txad108

**Published:** 2023-09-02

**Authors:** Felix M Njeri, Robert Patterson, Charles K Gachuiri, Elijah G Kiarie

**Affiliations:** Department of Animal Production, University of Nairobi, Nairobi, Kenya; Department of Animal Biosciences, University of Guelph, Guelph, Ontario, Canada N1G 2W1; CBS Bio Platforms Inc., Calgary, Alberta, Canada AB T2C 0J7; Department of Animal Production, University of Nairobi, Nairobi, Kenya; Department of Animal Biosciences, University of Guelph, Guelph, Ontario, Canada N1G 2W1

**Keywords:** broiler chickens, fiber degrading enzymes, solubilization, sunflower meal, wheat middlings

## Abstract

Pretreating fibrous feedstuffs with exogenous enzymes may improve their utilization in broiler chickens. Pretreatment of wheat middlings (**WM**) and sunflower meal (**SM**) with fiber degrading enzymes (**FDE**) was investigated for 1) in vitro solubilization of crude protein (**CP**) and fiber-degrading (experiment 1), and 2) apparent retention (**AR**) of CP, neutral detergent fiber (**NDF**), nitrogen corrected apparent metabolizable energy (**AMEn**), as well as the concentration of ceca digesta metabolites in broiler chickens (experiment 2). In experiment 1, WM was pretreated with FDE and SM with FDE ± protease and incubated in a shaker for 24 or 48 h at 40°C and 200 rpm. Samples were centrifuged, and the supernatant used for assay of sugars and organic acids and pellet processed for determination of apparent disappearance (**AD**) of dry matter (**DM**), fiber, and CP solubilization. In experiment 2, WM and SM were pretreated with FDE for 24 h, oven-dried, and incorporated in iso-caloric and iso-nitrogenous experimental diets. Diets were: 1) a corn–soybean meal positive control (**PC**); 2) PC plus untreated WM and SM (negative control, **NC**), and diets 3, 4, 5, and 6 test diets, in which the untreated WM and SM in NC were replaced with pretreated WM and SM at 25% (N25), 50% (N50), 75% (N75), and 100% (N100), respectively. Diets were prepared in mash form in two phases (starter, days 0 to 21 and finisher, days 22 to 42) and had TiO_2_ (0.3%) as an indigestible marker. A total of 288 Ross708 d-old male broiler chicks were placed in cages based on body weights (6 birds/cage) and allocated diets (*n* = 8). Birds had free access to feed and water. Samples of excreta for AR and AMEn, and of ceca digesta for the concentration of short-chain fatty acids (**SCFA**) were collected at the end of each phase. Pretreatment with FDE increased (*P* < 0.001) solubilization of CP, AD of NDF, and release of sugars and organic acids in the supernatant. The mixture of FDE and protease further increased (*P* < 0.001) CP solubilization in SM. Feeding pretreated WM and SM had a linear response (*P* ≤ 0.038) on AMEn, and gross energy (**GE**) (day 21) and a quadratic response (*P* < 0.05) on AR of components and AMEn (day 42) and concentration of total SCFA on day 42. On day 42, N25 and N50 had higher AR of DM, CP, NDF, and GE than N75 and N100. In conclusion, pretreatment of WM and SM with enzymes increased CP and fiber degradation. Incorporating moderate amounts (N25 and N50) of pretreated WM and SM in a corn–soybean meal diet fed to broiler chickens improved nutrient and energy utilization.

## Introduction

To reduce the cost of poultry production, agricultural byproducts such as rapeseed meal, sunflower meal (**SM**), wheat middlings (**WM**), and wheat bran are increasingly being used in diet formulations. However, these ingredients are high in anti-nutritional dietary fiber (Kithama et al., 2021; Lannuzel et al., 2022; Singh and Kim, 2021). Exogenous enzyme supplementation in broiler chicken feeds produced mixed results, with some researchers reporting improved performance (Sanchez et al., 2018; Agboola et al., 2015; Kiarie et al., 2017), while others reported no improvement (Mohammed et al., 2017; Olgun et al., 2018; Walters et al., 2018). These mixed results are because the exogenous enzymes usually encounter various limitations that reduce their efficacy (Ravindran, 2013). Factors such as the pH variability along the chicken’s gastrointestinal tract and short retention time of the feed in the gastrointestinal tract also contribute to the limitation of the enzyme responses (Ravindran, 2013). Furthermore, microbial sources of enzymes, variable dosing, mismatch between substrate and enzymes have been attributed to limiting our understanding of in vivo responses (Ndou et al., 2015: Kiarie et al., 2016; Bautil et al., 2021).

Further endeavors in advancing the utility of feed enzymes have focused on pretreating fibrous feedstuffs (Moran et al., 2016; Muchiri et al., 2023; Rho et al., 2018; 2020). Rho et al. (2020) pretreated corn distillers dried grains with solubles and WM with a xylanase, cellulose, and β-glucanase mixture. Pretreatment increased the apparent disappearance (**AD**) of neutral detergent fiber (**NDF**), and crude protein (**CP**) solubilization. Pretreating sunflower and canola meals with fiber degrading enzymes (**FDE**) increased concentration of soluble CP, amino acids, and mono sugars (**MS**) in the liquid phase relative to control (Tian et al., 2022; Ugolini et al., 2015). Similarly, Rahimi et al. (2020) pretreated broiler diets formulated to be deficient in calcium and phosphorous with a mixture of xylanase, β-glucanase, and 0.5% HCl with or without phytase and fed them to broiler chickens. The results showed an increase in feed intake, bone mineralization, and intestinal phosphorous bioavailability compared to control.

There are limited investigations on enzymatic pretreatment of fibrous ingredients and the impact of subsequent incorporation into a practical broiler chicken diet. The current study hypothesized that 1) in vitro pretreatment of SM and WM with fiber-degrading enzymes (FDE) would increase AD of fiber and CP and addition of protease in pretreating SM would increase CP solubilization. 2) Incorporating untreated SM and WM in a corn–soybean meal-based diet would reduce nutrients and energy utilization in broiler chickens. In converse, substituting untreated SM and WM with pretreated SM and WM will improve nutrient and energy utilization. Therefore, the effects of pretreating WM and SM with enzymes on 1) in vitro degradation of CP and fiber (experiment 1) and 2) apparent retention (**AR**) of CP, NDF, nitrogen-corrected apparent metabolizable energy (**AMEn**) and concentration of ceca digesta metabolites in broiler chickens (experiment 2) were investigated.

## Materials and Methods

### Experiment 1

#### Feed ingredients and treatments

The samples of WM were purchased from Floradale Feed Mill Limited (Floradale, ON, Canada) and expeller pressed SM from Persall Fine Foods Company (Waterford, ON, Canada), and were used without further processing. The WM pretreatments were 1) control; 2) control + FDE (xylanase + cellulase + β-glucanase), whereas the SM pretreatments were 1) control; 2) control + FDE (cellulase + β-glucanase + β-mannanase + pectinase); and 3) control + FDE + protease. The enzymes were mixed with materials at 1% (w/w) as per the supplier’s recommendations. The WM enzyme activities were 3,000 xylanase, 45,000 cellulase, and 40,000 β-glucanase U/g; the SM enzymes activities were 45,000 cellulase, 40,000 β-glucanase, 12,000 β-mannanase, 250,000 pectinase and 1,000,000 protease U/g (CBS Bio Platforms Inc, Calgary, AB, Canada).

#### Experimental procedures and sampling

The pretreatment of WM and SM was run in independent experiments with two time points (24 or 48 h) in four replicates for each pretreatment by time combination. For each combination, 50 g of either WM or SM was weighed and transferred into a 500-mL plastic container; 200 mL of distilled water was added, and the container was placed in an incubator shaker (Controlled Environment Incubator Shaker, New Brunswick Scientific, Enfield, CT). The incubator temperature was set at 40 °C, and the shaking speed was set to 200 rpm. The materials were allowed 15 min for the temperature to equilibrate, and 0.5 g of the respective enzymes were added. Containers were tightly capped throughout the incubation period and left for 24 or 48 h with continuous agitation. At the end of each time point, the pH was read immediately using a pH meter (Fisher Scientific) calibrated at three-points (4.0, 7.0, and 10.0). One replicate of each treatment was frozen entirely (the entire pretreatment mixture) at −20 °C until needed for analyses. To separate the supernatant and pellet, the remaining three replicates per treatment were centrifuged at 30,000 × 10 g and at 20 °C for 15 min (Rho et al., 2020). A 1-mL aliquot of the supernatant was frozen (−20 °C) until required for further analyses. The rest of the supernatant and pellet were weighed separately and frozen until required for further analyses.

### Experiment 2

The University of Guelph Animal Ethics and Research Committee approved the animal protocol (#4403). The birds were cared for in accordance with the Canadian Code of Practice for Animal Care and Use for Scientific Purposes (CCAC, 2009).

#### Feed ingredients, pretreatments, and diets

The same batch of ingredients that were used in experiment 1 were also used in experiment 2. WM and SM were pretreated in accordance with (Muchiri et al., 2023). Briefly, before diet preparation, each feedstuff was mixed with 1% of FDE, then with distilled water in a ratio of 1:2 (w/w) for feedstuff: water. The mixtures were then incubated for 24 h at 40 °C and oven-dried at 60 °C. The WM was pretreated with 60,000 xylanase, 8,000 β-glucanase, 38,000 cellulases U/g, and SM with 40,000 β-glucanase, 45,000 cellulases, 12,000 β-mannanase, and 25,000 pectinases U/g (CBS Bio Platforms Inc.). The diets were 1) a corn–soybean meal diet, positive control (**PC**); 2) a negative control (**NC**); PC plus both untreated SM (**USM**) and untreated WM (**UWM**); and diets 3, 4, 5, and 6 as test diets in which USM and UWM were replaced with FDE pretreated SM (**TSM**) and FDE pretreated WM (**TWM**) at 25% (N25), 50% (N50), 75% (N75), and 100% (N100), respectively (Table 1). The diets were formulated for two phases—starter (days 0 to 21) and finisher (days 22 to 42). The NC had a lower AME of 80 and 150 kcal/kg for the starter and finisher phase, respectively, compared to each phase PC. It was hypothesized that pretreatment of SM and WM would uplift energy utilization, bridging the energy deficit. This would be achieved by the capacity of FDE to hydrolyze non-starch polysaccharides (NSP) and encapsulate TSM and TWM cell walls to release trapped nutrients. All diets had titanium dioxide (0.3%) as an indigestible marker and were prepared in mash form.

**Table 1.** Composition of experimental diets, as-fed basis (experiment 2)

Ingredients, %	Starter (days 0 to 21)						Finisher (days 21 to 42)					
	PC	NC	NC25	NC50	NC75	NC100	PC	NC	NC25	NC50	NC75	NC100
Corn	63.6	53.2	53.2	53.2	53.2	53.2	66.2	48.9	48.9	48.9	48.9	48.9
Soybean meal 46%	24.0	16.4	16.4	16.4	16.4	16.4	23.1	12.8	12.8	12.8	12.8	12.8
Wheat middlings	—	10	7.5	5.0	2.5	—	—	18.5	13.9	9.3	4.6	—
Treated wheat middlings	—	—	2.5	5.0	7.5	10	—	—	4.6	9.3	13.9	18.5
Soy oil	3.0	3.5	3.5	3.5	3.5	3.5	3.3	4.5	4.5	4.5	4.5	4.5
Sunflower meal	—	7.5	5.6	3.8	1.9	—	—	8.0	6.0	4.0	2.0	—
Treated sunflower meal	—	—	1.9	3.8	5.6	7.5	—	—	2.0	4.0	6.0	8.0
Fish meal	1.5	1.5	1.5	1.5	1.5	1.5	—	—	—	—	—	—
Pork meal	5.0	5.0	5.0	5.0	5.0	5.0	5.0	5.0	5.0	5.0	5.0	5.0
l-Lysine HCL	0.32	0.44	0.44	0.44	0.44	0.44	0.13	0.3	0.3	0.3	0.30	0.30
dl-Methionine	0.15	0.15	0.15	0.15	0.15	0.15	0.20	0.20	0.20	0.20	0.20	0.20
Threonine	—	0.02	0.02	0.02	0.02	0.02	—	—	—	—	—	—
Tryptophan	—	0.01	0.01	0.01	0.01	0.01	—	0.01	0.01	0.01	0.01	0.01
Limestone	0.46	0.43	0.43	0.43	0.43	0.43	0.37	0.33	0.33	0.33	0.33	0.33
Monocalcium phosphate	0.74	0.63	0.63	0.63	0.63	0.63	0.61	0.42	0.42	0.42	0.42	0.42
Sodium chloride	0.40	0.44	0.44	0.44	0.44	0.44	0.32	0.32	0.32	0.32	0.32	0.32
Vitamin and trace minerals premix^1^	0.50	0.50	0.50	0.50	0.50	0.50	0.50	0.50	0.50	0.50	0.50	0.50
Titanium dioxide	0.30	0.30	0.30	0.30	0.30	0.30	0.30	0.30	0.30	0.30	0.30	0.30
Total	100	100	100	100	100	100	100	100	100	100	100	100
Calculated nutrient												
AMEn, kcal/kg	2,960	2,880	2,880	2,880	2,880	2,880	2,990	2,840	2,840	2,840	2,840	2,840
CP, %	20	19.5	19.5	19.5	19.5	19.5	18.7	18.1	18.1	18.1	18.1	18.1
SID Lys, %	1.22	1.19	1.19	1.19	1.19	1.19	0.99	0.96	0.96	0.96	0.96	0.96
SID Met, %	0.45	0.40	0.40	0.40	0.40	0.4	0.48	0.46	0.46	0.46	0.46	0.46
SID Met + Cys, %	0.75	0.75	0.75	0.75	0.75	0.75	0.77	0.72	0.72	0.72	0.72	0.72
SID Thr, %	0.88	0.67	0.67	0.67	0.67	0.67	0.86	0.84	0.84	0.84	0.84	0.84
SID Trp, %	0.20	0.16	0.16	0.16	0.16	0.16	0.18	0.18	0.18	0.18	0.18	0.18
Ca, %	0.96	0.96	0.96	0.96	0.96	0.96	0.84	0.84	0.84	0.84	0.84	0.84
Available *P*, %	0.48	0.48	0.48	0.48	0.48	0.48	0.42	0.42	0.42	0.42	0.42	0.42

PC, corn–soybean meal based positive control; NC, PC plus untreated sunflower meal (USM) and untreated wheat middling’s (UWM); N25, NC with USM and UWM replaced with 25% of treated sunflower meal (TSM) and 25% of treated wheat middling’s (TWM), respectively; N50, NC with USM and UWM replaced with 50% of TSM and 50% of TWM, respectively; N75, NC with USM and UWM replaced with75% TSM and 75% TWM, respectively; N100, NC with USM and UWM replaced with 100% TSM and 100% of TWM, respectively.

^1^Provided per kilogram of premix: vitamin A (retinol), 880 KIU; vitamin D3 (cholecalciferol), 330 KIU; vitamin E, 4,000 IU; vitamin K3 (menadione), 330 mg; vitamin B1 (thiamin), 400 mg; vitamin B2 (riboflavin), 800 mg; vitamin B3 (niacin), 5,000 mg; vitamin B5 (pantothenic acid), 1,500 mg; vitamin B6 (pyridoxine), 300 mg; vitamin B9 (folic acid), 100 mg; vitamin B12 (cyanocobalamin), 1,200 mcg; biotin, 200 mcg; choline, 60,000 mg; Fe, 6,000 mg; Cu, 1,000 mg; I, 1 mg, Se, 30 mg.

#### Experimental procedures and sampling

A total of 288 Ross 708 d-old male broiler chicks were placed in cages (6 birds/cage) based on body weight and allocated to 6 diets with 8 replicates per diet in a completely randomized design. Birds had free access to feed and water. Excreta were collected per cage from days 18 to 20 for the starter phase and from days 39 to 41 for the finisher phase. The excreta samples were pooled per cage and frozen until needed for analysis in each phase.

### Sample Processing and Chemical Analyses

The frozen whole pretreatment mixture, as well as the separated supernatant and pellet, were all freeze-dried. The whole mixture, together with untreated SM and untreated WM, was then ground using a coffee grinder (CBG5 Smart Grind; Applica Consumer Products, Inc., Shelton, CT), and subsequently submitted for CP, soluble CP (**SCP**), acid detergent CP (**ADCP**), neutral detergent CP (**NDCP**), NDF, acid detergent fiber (ADF), lignin, and mineral analyses to a commercial laboratory (SGS Canada Inc., Guelph, ON, Canada). The dried pellet samples were weighed and analyzed in triplicate for DM, NDF, and CP. The DM was analyzed using method 930.15 (AOAC, 2004), and the NDF was determined using an ANKOM 200 fiber analyzer (ANKOM Technology, Macedon, NY), as described by van Soest et al. (1991). Nitrogen was determined using the LECO machine (LECO Corporation, St. Joseph, MI) method 968.06 (AOAC International, 2005), and CP values were derived by multiplying the *N* value by 6.25. The soluble CP was analyzed as per the method described by Roe et al. (1990). The NDCP and ADCP were determined by analyzing nitrogen in the respective residues of the NDF and ADF determinations. The AOAC method 965.09 (AOAC, 2006) was used to determine the concentration of calcium, phosphorus, potassium, magnesium, copper, manganese, zinc, and iron.

Using high-performance liquid chromatography (**HPLC**; Agilent 1100 Series, Agilent Technologies), the supernatant was used to determine the concentrations of MS and organic acids (Leung et al., 2018; Rho et al., 2020). Samples were thawed, vortexed, and centrifuged for 15 min. The fat layer was vacuumed away. A sample of 160 µL was diluted × 20 with 0.005 N sulfuric acid, filtered with a 13-mm syringe filter, and transferred to the HPLC vials. The analytes were separated using a 30 × 7.8-mm, 8-µm RezexTM ROA-Organic Acid H + (8%) column (Phenomenex, Torrance, CA). The HPLC parameters were as follows: 60 °C column temperature, 20 µL injection volume, 35 °C refractive index detector temperature, 0.5 mL/min 0.005 N sulfuric acid mobile phase velocity, and a cycle time of 45 min. The retention times for glucose, xylose, arabinose, lactic, acetic, propionic, isobutyric, and butyric were, respectively, 12.4, 13.2, 13.7, 16.5, 19.6, 22.8, 25.5, and 28.0 min.

The excreta samples were thawed and weighed before and after oven drying at 65 °C to determine moisture content. A coffee grinder was used to grind dried excreta and diets (CBG5 Smart Grind; Applica Consumer Products, Inc.). The DM, CP, and NDF levels in excreta and diet samples were measured as previously described. Gross energy (**GE**) in excreta and diet samples was determined using an adiabatic bomb calorimeter (IKA Calorimeter System C 6000; IKA Works, Wilmington). The titanium content in diets and excreta was analyzed according to (Myers et al., 2004). Samples of unground diets were further analyzed for particle size using an RO-TAP Sieve Shaker (model RX-30 E; W.S. Tyler, Mentor, OH).

### Calculation and Statistical Analyses

The following equation was used to calculate AD of components as described by Rho et al. (2020).


$$\begin{array}{lll} AD=\; & [\left(\left(concentration\;before\;pretreatment\right)-\left(concentration\;after\;pretreatment\right)\right)\; & /\left(\left(concecentration\;before\;pretreatment\right)\right)] \end{array}$$

The AR of components was calculated as described by Kiarie et al. (2014).


$$\begin{array}{l} AR=\left[\frac{\left(NT/Ti\right)diet-\left(NT/Ti\right)excreta}{\left(NT/Ti\right)diet}\right] \end{array}$$

where NT/Ti is the ratio of component of interest and titanium in the diet, whereas NT/Ti is the ratio of component of interest and titanium in excreta. The components could be DM, NDF, CP, GE, or AMEn.

Apparent metabolizable energy was calculated using the following equation as described by Mwaniki and Kiarie (2019):


$$\begin{array}{l} AME\left(\frac{kcal}{kg}/DM\right)=\frac{\left[(AR\;of\;GE)\times (GE\;content\;of\;the\;diet)\right]}{100} \end{array}$$

Nitrogen-corrected apparent metabolizable energy (AMEn) was calculated using the equation below, as described by Mwaniki and Kiarie (2019):


$$\begin{array}{l} AMEn\left(\left(\frac{kcal}{kg}\right)/DM\right)=AME\;\;\;-(8.22\times ARN) \end{array}$$

where AME is the apparent metabolizable energy on a dry matter basis and ARN of apparent retention of nitrogen.

The container was the experimental unit for experiment 1, and data were subjected to GLIMMIX procedures of SAS 9.4. The model had treatments, time, and their interactions as fixed factors.


$$\begin{array}{l} Y_{ijk}=\;\mu +\;b_{i}c_{j}\;+\;bc_{ij}+\;\varepsilon_{ijk} \end{array}$$

where *Y*_*ijk*_ was observation recorded such xylose, arabinose; μ_*i*_ = overall population mean; *b*_*j*_ = treatment effect; *c* = time effect; *bc*_*ij*_ = interaction effect between treatment and time; ɛ_*ijk*_ = random error effect associated with observation recorded.

The Tukey significance difference test was used to separate the significant means (*P* < 0.05).

For experiment 2, the cage was the experimental unit. Outliers were removed using the PROC UNIVARIATE in SAS. Any value above or below the mean ± 3 standard deviations was identified and removed as an outlier. The analysis model had diet as a fixed factor.


$$\begin{array}{l} Y_{ij}=\;\mu +\;\alpha +\;\varepsilon_{ij} \end{array}$$

where *Y*_*ij*_ was observations recorded such AR of CP, NDF, etc.; μ = overall population means; α = treatment effect; ɛ_*ij*_ = random error effect associated with observations recorded.s

The Tukey significance difference test was used to separate the significant means (*P* < 0.05). Pre-planned contrast was used for PC vs. NC and incorporation of treated feedstuffs in NC was evaluated for linear and quadratic responses.

## Results

### Experiment 1

The concentration of SCP in control WM was greater at 24 and 48 h than at 0 h (Table 2). In contrast, the concentration of SCP in control SM was greater at 0 h than at 24 and 48 h (Table 2). When compared to the control WM, FDE-pretreated WM had a higher concentration of SCP in the supernatant, lignin, and crude fat in the pellet, and lower levels of ADF, NDF, and NDCP at 24 and 48 h, respectively. At 24 and 48 h, the control SM contained less SCP but more ADCPs, NDCP, and NDF than the FDE-pretreated SM or FDE plus protease.

**Table 2.** Analyzed chemical composition (g/kg) of wheat middlings and sunflower meal pretreated with fiber degrading enzymes (FDE), on dry matter basis (experiment 1)

	Wheat middlings^1^					Sunflower meal^2^						
Time, h	0^3^	24		48		0	24			48		
Treatments	Control	Control	FDE	Control	FDE	Control	Control	FDE	FDEP^4^	Control	FDE	FDEP^4^
Crude protein	204.2	207.6	222.5	215.0	230.5	315.2	315.4	335.2	325.9	290.8	338.8	337.2
Soluble protein	90.70	128.7	161.6	137.8	190.6	218.9	152.6	280.0	276.8	169.6	295.3	302.5
Acid detergent protein	5.70	6.50	6.10	7.40	6.60	8.60	15.80	11.90	10.50	12.20	9.80	9.10
Neutral detergent protein	44.00	40.60	27.90	43.90	36.20	26.10	79.00	28.80	20.50	50.10	32.20	20.50
Acid detergent fiber	136.4	135.4	121.4	144.8	118.9	208.5	347.3	254.5	254.0	255.5	202.2	208.2
Neutral detergent fiber	406.5	407.7	312.4	423.4	304.2	313.7	479.7	347.9	346.3	347.8	272.4	277.8
Lignin	36.80	57.80	66.20	47.20	61.10	78.30	179.8	210.3	120.9	167.8	155.3	166.7
Crude fat	29.80	43.40	49.10	45.10	55.30	132.8	128.1	133.8	116.9	130.6	130.7	125.1
Starch	188.6	171.4	175.6	167.0	187.4	10.60	6.40	7.70	7.10	6.40	7.80	8.90
pH	6.25	—	—	—		6.13	—	—	—	—	—	—

^1^About 50 g of wheat middlings were mixed with 200 mL of distilled water and 0.5 g of FDE mixture (3,000 xylanase, 45,000 cellulase, and 40,000 β-glucanase U/g).

^2^About 50 g of sunflower meal were mixed with 200 mL of distilled water and 0.5 g of FDE mixture (45,000 cellulase, 40,000 β-glucanase, 40,000 β-glucanase, 250,000 pectinase and 1,000,000,000 proteases U/g) and incubated at 40 °C inside an incubator shaker (Controlled Environment Incubator Shaker, New Brunswick Scientific) with continuous agitation of 200 rpm for 24 or 48 h.

^3^Time 0 h corresponded to untreated sample.

^4^FDE plus protease.

The effects of WM and SM pretreatment with FDE on AD of DM, NDF, CP, and pH are shown in Table 3. For WM, there was a treatment effect on AD of DM, NDF, and CP (*P* < 0.001) with FDE showing higher (*P* < 0.001) values than the control. There were no (*P* > 0.05) treatment or treatment and time interaction effects on WM pH. However, the time effect was such that pH was greatest (*P* = 0.003) at 24 h. For SM, there was no time effect or treatment and time interaction effects (*P* > 0.05) on AD of NDF. There were treatment and time interaction effects (*P* ≤ 0.046) on AD of DM and CP. The AD of DM was such that it was highest in FDE and FDE plus protease (**FDEP**) both at 24 and 48 h than the control, respectively. Also, the AD of CP was highest in FDE and FDEP at 48 h. There was treatment and time interaction on SM pH, with FDE at 24 and 48 h and FDEP at 48 h being the lowest (*P* = 0.001).

**Table 3.** Effects of pretreating wheat middlings and sunflower meal with fiber degrading enzymes on apparent disappearance of dry matter (DM), neutral detergent fiber (NDF) and crude protein (CP) (g/kg), and pH (experiment 1)

	Wheat middlings^1^					Sunflower meal^2^				
Item	Time, h	DM	NDF	CP	pH	Time, h	DM	NDF	CP	pH
Treatment										
Control		353.8^b^	403.5^b^	474.2^b^	3.64		205.3^b^	34.0^b^	324.2^c^	4.25^a^
FDE		470.2^a^	604.1^a^	595.3^a^	3.60		339.8^a^	267.4^a^	409.7^b^	3.80^c^
FDEP		—	—	—			345.9^a^	258.5^a^	502.7^a^	3.92^b^
SEM		8.73	14.38	8.68	0.02		6.36	19.01	12.31	0.03
Time, h										
24		406.2	501.3	525.2	3.68^a^		295.0	188.8	349.1^b^	4.01
48		417.8	506.3	544.3	3.56^b^		299.1	184.4	475.4^a^	3.97
SEM		8.73	14.38	8.68	0.02		5.19	15.52	10.05	0.02
Treatment										
Control	24	347.9^b^	403.6^b^	465.0^b^	3.72^b^	24	199.2^b^	53.1^b^	251.7^d^	4.18^ba^
Control	48	359.6^b^	403.4^b^	483.4^b^	3.57^a^	48	211.5^b^	14.9^b^	396.8^bc^	4.33^a^
FDE	24	464.4^a^	599.0^a^	585.4^a^	3.65^b^	24	327.6^a^	264.9^a^	328.0^dc^	3.81^c^
FDE	48	476.0^a^	609.1^a^	605.1^a^	3.56^a^	48	352.1^a^	269.9^a^	491.5^a^	3.79^c^
FDEP	—	—	—	—	—	24	358.1^a^	248.6^a^	467.6^ba^	4.03^b^
FDEP	—	—	—	—	—	48	333.7^a^	268.5^a^	537.8^a^	3.80^c^
SEM		12.35	20.34	12.27	0.03		9.00	26.89	17.41	0.04
*P*-Value										
Treatment		<0.001	<0.001	<0.001	0.175		<0.001	<0.001	<0.001	<0.001
Time		0.373	0.813	0.159	0.003		0.589	0.844	<0.001	0.330
Treatment * time		1.000	0.807	0.959	0.341		0.047	0.549	0.046	0.001

^1^About 50 g of wheat middlings were mixed with 200 mL of distilled water and 0.5 g of FDE mixture (3,000 xylanase, 45,000 cellulase and 40,000 β-glucanase U/g).

^2^About 50 g of sunflower meal were mixed with 200 mL of distilled water and 0.5 g of FDE mixture (45,000 cellulase, 40,000 β-glucanase, 40,000 β-glucanase, 250,000 pectinase, and 1,000,000,000 protease U/g) and incubated at 40 °C inside an incubator shaker (Controlled Environment Incubator Shaker, New Brunswick Scientific) with continuous agitation of 200 rpm for 24 or 48 h.

Values within a column without a common superscript differ significantly by LS means at *P* < 0.05.

There was a treatment effect on the concentration of xylose, arabinose, and glucose in WM, with FDE having higher concentrations than the control (*P* < 0.001) (Table 4). The concentrations of xylose and arabinose in WM showed no time or treatment and time interaction effects (*P* > 0.05). There was a time effect (*P* = 0.004) on glucose concentration, with 24 h being greater than 48h, but there were no time and treatment interaction effects (*P* > 0.05) on the glucose concentration. There was no interaction between treatment and time on the concentration of total sugars in WM (*P* > 0.05). However, treatment and time effects (*P* ≤ 0.028) were observed on total sugars in pretreated WM, with FDE being higher than the control and 24 h being greater than 48 h. Lactic acid concentration had treatment and time effects (*P* = 0.002), but no treatment and time interaction effect (*P* > 0.05). There was no treatment, no time, and interaction effects (*P* > 0.05) on the concentration of acetic, isobutyric, and butyric acids. There were no treatment and time interaction (*P* > 0.05) on the concentration of propionic acid, but the treatment effect (*P* < 0.001) was such that FDE had a higher concentration than the control. There was no time and treatment interaction on the concentration of total organic acid (*P* > 0.05). However, treatment and time effects (*P* < 0.001) were such that FDE and 48 h had higher total organic acids concentrations than the control and 24 h, respectively.

**Table 4.** Effects of pretreating wheat middlings with fiber degrading enzymes (FDE) on concentration (nmol/kg) of mono-sugars (MS) and organic acids in the supernatant over time (experiment 1)

		Mono-sugars				Organic acids					
Item	Time, h	Xylose	Arabinose	Glucose	Total	Lactic	Acetic	Propionic	Isobutyric	Butyric	Total
Treatment^1^											
Control		44.24^b^	4.54^b^	11.34^b^	60.11^b^	375.9^b^	32.94	7.59^b^	2.89	6.8	426.1^b^
FDE		170.35^a^	32.41^a^	36.26^a^	239.0^a^	392.7^a^	40.16	16.49^a^	2.38	7.22	458.96^a^
SEM		2.8	0.43	3.14	5.62	3.31	2.51	1.49	0.21	1.52	3.51
Time, h											
24		109.06	18.23^a^	31.10a	159.0^a^	341.8^b^	34.55	11.74	2.52	6.11	396.8^b^
48		105	18.71^b^	16.50b	140.2^b^	426.7^a^	38.54	12.33	2.76	7.91	488.3^a^
SEM		2.8	0.43	3.14	5.6	3.31	2.51	1.42	0.21	1.52	3.51
Treatment^1^											
Con	24	46.14^b^	3.91^b^	17.59^bc^	67.63^b^	330.0^c^	33.28	6.37^c^	2.88	7.39	379.9^c^
Con	48	42.34^b^	5.17^b^	5.09^c^	52.59^b^	421.7^a^	32.59	8.82^bc^	2.9	6.21	472.2^b^
FDE	24	173.1^a^	32.55^a^	44.61^a^	250.3^a^	353.6^b^	35.83	17.12^a^	2.16	4.84	413.6^c^
FDE	48	167.6^a^	32.26^a^	27.91^ba^	227.8^a^	431.8^a^	44.88	15.85^ba^	2.61	9.61	504.3^a^
SEM		3.97	0.61	4.45	0.01	4.68	3.55	2.01	0.22	0	4.96
*P*-value											
Treatment		<0.001	<0.001	<0.001	<0.001	0.002	0.056	<0.001	0.107	0.846	<0.001
Time		0.254	0.436	0.004	0.028	<0.001	0.275	0.772	0.443	0.412	<0.001
Treatment * time		0.830	0.219	0.642	0.643	0.163	0.203	0.364	0.484	0.181	0.876

FDE: fiber degrading enzymes.

^1^About 50 g of wheat middlings were mixed with 200 mL of distilled water and 0.5 g of FDE mixture (3,000 xylanase, 45,000 cellulase and 40,000 β-glucanase U/g) and incubated at 40 °C inside an incubator shaker (Controlled Environment Incubator Shaker, New Brunswick Scientific) with continuous agitation of 200 rpm for 24 or 48 h.

Values within a row without a common superscript differ significantly by LS means at *P* < 0.05.

The concentration of xylose in the SM supernatant showed time and treatment interaction effects (*P* = 0.015), with FDE and FDEP being greater than the control both at 24 and 48 h (Table 5). No treatment and time interaction (*P* > 0.05) was observed for the concentration of arabinose in SM. A treatment effect (*P* < 0.001) was such that FDE and FDEP had higher arabinose concentrations than the control. The glucose concentration had a treatment and time interaction effect (*P* < 0.001), with the highest concentration being observed in FDEP and 48 h. There were no treatment and time interaction or time effects (*P* > 0.05) on the concentration of total sugar; the treatment effect (*P* < 0.001) was that FDE and FDEP had higher than the control. There was treatment and time interaction (*P* < 0.001) on the concentrations of lactic and propionic acid, with FDEP being highest at 48 h. The concentration of acetic acid had treatment and time interaction effect (*P* = 0.002), with FDEP both at 24 h and 48 h being the highest. There were no treatment effects on the concentration of isobutyric (*P* > 0.05). There were no time or interaction effects (*P* > 0.05) on butyric acid concentration; however, a treatment effect (*P* < 0.001) was such that FDE and FDEP had higher concentrations than the control. Total organic acid concentration had a treatment and time interaction effect (*P* < 0.001), with FDEP at 48 h having the highest concentration.

**Table 5.** Effects of pretreating sunflower meal with fiber degrading enzymes (FDE) on concentration (nmol/kg) of mono sugars and organic acids in the supernatant over time (experiment 1)

Item	Time, h	Xylose	Arabinose	Glucose	Total sugars	Lactic	Acetic	Propionic	Isobutyric	Butyric	Total organic acids
Treatment^1^											
Control		10.45^b^	2.20b	2.87^c^	15.52^c^	246.2^b^	17.68^b^	8.75^b^	4.14	8.36^b^	285.13^b^
FDE		39.83^a^	23.57a	23.30^b^	86.70^a^	384.1^a^	44.23^a^	7.66^b^	3.85	20.81^a^	460.65^a^
FDEP		36.24^a^	28.69a	24.92^a^	89.84^a^	377.3^a^	48.62^a^	29.81^a^	4.02	20.97^a^	480.70^a^
SEM		2.93	2.52	0.28	2.94	7.04	2.96	0.39	0.23	0.89	6.13
Time, h											
24		31.37	19.7	14.49^b^	65.56	306.9^b^	33.49	14.58^b^	4.20	16.08	375.22^b^
48		26.3	16.6	19.57^a^	62.47	364.9^a^	40.19	16.23^a^	3.80	17.34	442.43^a^
SEM		2.40	2.06	0.23	2.38	5.75	2.42	0.32	0.19	0.73	5.00
Treatment^1^											
Control	24	10.47^c^	3.9	2.16^d^	16.53^b^	251.5^d^	13.37^d^	9.72^c^	4.48	7.78^b^	286.87^d^
Control	48	10.43^c^	0.5	3.58^d^	14.51^b^	240.9^d^	21.99^dc^	7.77^dc^	3.81	8.94^b^	283.40^d^
FDE	24	49.71^a^	24.06^a^	19.34^c^	93.11^a^	365.7^b^	33.26^bc^	7.15^d^	4.13	19.77^a^	430.05^c^
FDE	48	29.94^b^	23.09^ab^	27.26^a^	80.29^a^	402.5^b^	55.19^a^	8.18^dc^	3.57	21.84^a^	491.24^b^
FDEP	24	33.93^ba^	31.15^a^	21.98^b^	87.06^a^	303.3^c^	53.84^a^	26.87^b^	4.00	20.70^a^	408.74^c^
FDEP	48	38.54^ba^	26.21^a^	27.87^a^	92.62^a^	451.3^a^	43.39^ba^	32.74^a^	4.03	21.24^a^	552.67^a^
SEM		4.14	3.57	0.4	4.16	9.96	4.18	0.55	0.33	1.26	8.60
*P*-value											
Treatment		<0.001	<0.001	<0.001	<0.001	<0.001	<0.001	<0.001	0.668	<0.001	<0.001
Time		0.145	0.295	<0.001	0.366	<0.001	0.059	0.001	0.145	0.229	<0.001
Treatment * time		0.015	0.854	<0.001	0.099	<0.001	0.002	<0.001	0.528	0.833	<0.001

FDEP, fiber degrading enzymes plus proteases.

^1^About 50 g of sunflower meal were mixed with 200 mL of distilled water and 0.5 g of FDE mixture (45,000 cellulase, 40,000 β-glucanase, 40,000 β-glucanase, 250,000 pectinase and 1,000,000,000 protease U/g) and incubated at 40 °C inside an incubator shaker (Controlled Environment Incubator Shaker, New Brunswick Scientific) with the continuous agitation of 200 rpm for 24 or 48 h.

Values within a row without a common superscript differ significantly by LS means at *P* < 0.05.

### Experiment 2

The particle size (geometrical mean diameter ± standard deviation) of PC, NC, N25, N50, N75, and N100 for the starter diets was 862 ± 1.9 µm, 859 ± 1.8 µm, 763 ± 2.0 µm, 681 ± 2.1 µm, 843 ± 1.7 µm, and 831 ± 1.8 µm, respectively. The corresponding values for finisher diets were 678 ± 2.1 µm, 735 ± 1.9 µm, 864 ± 1.7 µm, 785 ± 1.8 µm, 785 ± 1.8 µm, and 857 ± 1.8 µm, respectively (Table 6). Comparatively, the NC diet had a higher concentration of crude fat and NDF than PC, and the PC had a higher concentration of starch (Table 6). Within phase, the concentration of CP was comparable between diets. The NDF concentration was 1.6- and 2-fold higher than PC in the starter and finisher phases, respectively. However, the concentration of NDF was comparable between NC and test diets.

**Table 6.** Analyzed composition of experimental diets, as-fed basis (experiment 2)

Treatments^1^	Starter (days 0 to 21)						Finisher (days 21 to 42)					
	PC	NC	N25	N50	N75	N100	PC	NC	N25	N50	N75	N100
Dry matter, %	88.5	90.3	88.5	88.4	88.4	88.4	88.4	88.3	89.3	88.5	88.1	87.7
Gross energy, kcal/kg	4,067	4,253	4,133	4,207	4,181	4,223	4,070	4,232	4,327	4,212	4,251	4,241
Starch, %	39.8	35.4	32.6	31.9	31.6	34.0	41.6	33.1	32.9	31.9	31.3	30.4
Crude protein, %	20.7	20.0	20.5	19.9	19.8	20.2	19.4	18.3	18.9	19.0	19.2	18.9
Crude fat, %	4.02	6.05	7.15	7.16	6.14	7.73	4.93	7.94	8.08	8.05	8.28	8.31
Neutral detergent fiber, %	7.41	11.8	12.2	11.5	11.8	11.3	7.37	14.9	14.8	14.8	12.1	13.1
Ash, %	5.38	5.75	5.82	5.84	5.87	5.86	4.65	5.22	5.03	5.29	5.21	5.40
Calcium, %	0.85	0.79	0.83	0.82	0.77	0.77	0.66	0.57	0.57	0.61	0.55	0.62
Phosphorous, %	0.68	0.73	0.75	0.74	0.72	0.76	0.56	0.68	0.7	0.7	0.68	0.71
GSD ± STDEV, µm	862 ± 1.9	859 ± 1.8	763 ± 2.0	681 ± 2.1	843 ± 1.7	831 ± 1.8	678 ± 2.1	735 ± 1.9	864 ± 1.7	785 ± 1.8	785 ± 1.8	857 ± 1.8

^1^PC, corn–soybean based positive control; NC, PC plus untreated sunflower meal (USM) and untreated wheat middling’s (UWM); N25, NC with USM and UWM replaced with 25% of treated sunflower meal (TSM) and 25% of treated wheat middling’s (TWM), respectively; N50, NC with USM and UWM replaced with 50% of TSM and 50% of TWM, respectively; N75, NC with USM and UWM replaced with75% TSM and 75% TWM, respectively; N100, NC with USM & UWM replaced with 100% TSM and 100% of TWM, respectively.

GSD ± STDEV (µm), geometrical mean diameter ± standard deviation (µm).

On day 21, the AR of DM and GE in broiler chickens fed either NC or PC diets was similar (*P* > 0.05; Table 7). However, birds that were fed PC had lower (*P* < 0.001) AR of AMEn, CP and NDF than those fed the NC diet. On day 21, birds fed N25, N50, and N100 retained less amounts of (*P* < 0.001) DM, and GE than birds fed PC. However, they retained more NDF (*P* < 0.001) than PC. The AMEn responded linearly (*P* < 0.001) from N25 to N75 with a drastic drop at N100. There was a (*P* < 0.001) treatment effect on the excreta moisture on day 21. There was a linear decrease on day 21 excreta moisture with inclusion of pretreated SM and WM. On day 42, the AR of DM, GE, and AMEn in NC were lower (*P* < 0.001), while NDF was higher (*P* < 0.001) than PC. The AR of DM, CP, GE, NDF and AMEn concentrations in test diets had a quadratic response (*P* < 0.001). The AR of AMEn, CP, and NDF in birds fed the test diets was higher than PC- and NC-fed birds. The excreta moisture concentration of NC was lower (*P* < 0.001) than PC, while the test diets had a quadratic response (*P* < 0.001) with N100 being the lowest.

**Table 7.** Apparent retention of components, apparent metabolizable energy corrected for nitrogen (AMEn, mcal/kg DM), and excreta moisture content (%) in broiler chickens fed corn–soybean meal-based diets with pretreated sunflower meal and wheat middlings (experiment 2)

	Treatments^1^								*P*-value		
	PC	NC	N25	N50	N75	N100	SEM	Overall	PC *vs.* NC	Linear	Quadratic
Day 21											
Dry matter	69.49^ba^	69.52^ba^	67.54^c^	68.73^bc^	70.73^a^	69.02^b^	0.47	<0.001	0.326	0.376	0.935
AMEn	3.45^dc^	3.50^bc^	3.41^d^	3.54^ba^	3.60^a^	3.57^ba^	0.02	<0.001	<0.001	<0.001	0.678
Crude protein	60.27^c^	65.18^a^	61.86^bc^	63.47^ba^	64.22^ba^	62.18^bc^	0.80	<0.001	0.021	0.073	0.928
Neutral detergent fiber	21.52^c^	35.39^b^	44.28^a^	41.60^a^	41.60^a^	34.95^b^	1.43	<0.001	<0.001	0.198	<0.001
Gross energy	71.89^ba^	71.64^ba^	70.01^c^	71.12^bc^	72.84^a^	71.36^bac^	0.51	<0.001	0.315	0.038	0.384
Excreta moisture	72.58^a^	72.04^ba^	70.78^bac^	70.09^bac^	69.48^bc^	68.04^c^	1.00	<0.001	<0.001	<0.001	0.334
Day 42											
Dry matter	71.16^ba^	65.46^e^	72.49^a^	69.86^bc^	67.91^d^	68.94^dc^	0.51	<0.001	<0.001	0.047	<0.001
AMEn	3.52^cd^	3.46^d^	3.77^a^	3.57^cb^	3.59^b^	3.63^b^	0.02	<0.001	<0.001	0.003	<0.001
Crude protein	58.86^c^	59.49^c^	65.76^a^	61.65^b^	60.15^cb^	59.50^c^	0.58	<0.001	0.277	<0.001	<0.001
Neutral detergent fiber	16.77^c^	35.25^b^	49.16^a^	47.55^a^	32.25^b^	35.70^b^	1.35	<0.001	<0.001	<0.001	<0.001
Gross energy	74.43^b^	69.60^d^	76.05^a^	72.87^c^	72.02^c^	72.69^c^	0.50	<0.001	0.001	0.074	<0.001
Excreta moisture	66.89^a^	64.48^c^	66.65^ba^	63.98^dc^	64.88^bc^	62.31^d^	0.66	<0.001	<0.001	<0.001	0.003

^1^PC, corn–soybean based positive control; NC, PC plus untreated sunflower meal (USM) and untreated wheat middling’s (UWM); N25, NC with USM and UWM replaced with 25% of treated sunflower meal (TSM) and 25% of treated wheat middling’s (TWM), respectively; N50, NC with USM and UWM replaced with 50% of TSM and 50% of TWM, respectively; N75, NC with USM and UWM replaced with75% TSM and 75% TWM, respectively; N100, NC with USM and UWM replaced with 100% TSM and 100% of TWM, respectively.

AMEn, nitrogen corrected apparent metabolizable energy.

Values within a row without a common superscript differ significantly by LS means at *P* < 0.05.

On day 21, the concentrations of MS and SCFA in ceca digesta were unaffected by dietary treatments (*P* ≥ 0.302), except for propionic acid (*P* = 0.008), which was lower in birds fed the test diets compared to PC and NC birds (Table 8). Test diets showed quadratic response (*P* = 0.046) on ceca digesta concentration of propionic acid, with N75 being the lowest. There were no differences (*P* > 0.05) between PC vs. NC on the MS concentration, except for arabinose, which was higher (*P* = 0.019) in PC on day 42. On day 42, birds fed the test diets had a linear decrease (*P* = 0.001) on the ceca digesta concentrations of arabinose. On day 42, there was no difference (*P* > 0.05) in SCFA concentration between PC and NC, except for propionic acid concentration (*P* = 0.039), which was higher in PC vs. NC. The test diets linearly decreased the concentration of lactic acid (*P* < 0.001) while linearly increased the concentration of isobutyric acid (*P* < 0.001). The concentration of valeric acid had a quadratic response (*P* = 0.023) and N100 had the lowest (*P* = 0.046) total SCFA concentration.

**Table 8.** Concentration of sugars and short-chain fatty acids (SCFA) (µmol/g) in the ceca digesta of broiler chickens fed corn-soybean meal-based diets with pretreated sunflower meal and wheat middlings (experiment 2)

	Treatments^1^							*P*-value			
Item	PC	NC	N25	N50	N75	N100	SEM	Overall	PC *vs.* NC	Linear	Quadratic
Day 21											
Glucose	0.38	0.62	0.28	0.65	0.53	0.79	0.24	0.708	0.462	0.465	0.439
Xylose	0.20	0.20	0.21	0.27	0.22	0.29	0.06	0.680	0.967	0.209	0.909
Arabinose	0.24	0.24	0.14	0.21	0.15	0.18	0.05	0.444	0.981	0.354	0.452
Total sugars	0.39	0.39	0.29	0.26	0.35	0.53	0.11	0.425	0.991	0.231	0.062
Lactic	4.44	3.69	3.03	3.98	3.09	2.93	0.44	0.102	0.217	0.222	0.559
Acetic	8.76	9.32	6.91	7.96	7.09	7.89	0.96	0.333	0.658	0.275	0.130
Propionic	1.22^a^	1.27^a^	1.06^ba^	1.09^ba^	0.87^b^	1.05^ba^	0.07	0.008	0.606	0.005	0.046
Isobutyric	0.40	0.41	0.43	0.42	0.38	0.48	0.04	0.384	0.709	0.453	0.251
Butyric	1.94	1.80	1.55	1.51	1.46	1.52	0.28	0.782	0.711	0.398	0.512
Isovaleric	0.27	0.31	0.24	0.32	0.30	0.35	0.05	0.766	0.511	0.522	0.574
Valeric	0.44	0.39	0.29	0.32	0.36	0.32	0.06	0.384	0.475	0.723	0.447
Total SCFA^*^	14.78	17.06	12.89	15.33	13.33	14.2	1.42	0.302	0.231	0.146	0.181
Day 42											
Glucose	0.65	0.59	0.69	0.38	0.36	0.28	0.16	0.132	0.782	0.033	0.858
Xylose	0.20	0.14	0.21	0.17	0.20	0.20	0.03	0.580	0.193	0.243	0.575
Arabinose	0.38^a^	0.27^ba^	0.26^ba^	0.21^b^	0.19^b^	0.16^b^	0.03	<0.001	0.019	0.005	0.959
Total sugars	0.93	0.69	0.96	0.76	0.70	0.64	0.16	0.615	0.289	0463	0372
Lactic	0.96^a^	0.86^a^	0.86^ba^	0.72^ba^	0.70^ba^	0.57^b^	0.06	0.002	0.318	<0.001	0.601
Acetic	10.75	9.27	10.73	9.99	9.10	9.00	0.59	0.130	0.083	0.282	0.172
Propionic	1.58^a^	1.33^ba^	1.16^b^	1.45^ba^	1.15^b^	1.13^b^	0.08	0.001	0.039	0.101	0.290
Isobutyric	0.45^ba^	0.39^b^	0.42^ba^	0.48^ba^	0.51^a^	0.50^a^	0.02	0.003	0.065	<0.001	0.194
Butyric	1.69	1.89	2.63	1.87	1.62	1.6	0.29	0.141	0.632	0.112	0.381
Isovaleric	0.28	0.25	0.25	0.27	0.29	0.26	0.09	0.993	0.671	0.849	0.864
Valeric	0.36	0.33	0.45	0.48	0.32	0.36	0.04	0.046	0.641	0.603	0.023
Total SCFA^*^	16.00^ba^	14.18^ba^	16.46^a^	15.12^ba^	13.30^b^	13.22^b^	0.91	0.046	0.140	0.089	0.138

^*^Summation of lactic, acetic, propionic, iso-butyric, butyric, iso-valeric, and valeric.

^1^PC, corn–soybean based positive control; NC, PC plus untreated sunflower meal (USM) and untreated wheat middling’s (UWM); N25, NC with USM and UWM replaced with 25% of treated sunflower meal (TSM) and 25% of treated wheat middling’s (TWM), respectively; N50, NC with USM and UWM replaced with 50% of TSM and 50% of TWM, respectively; N75, NC with USM and UWM replaced with75% TSM and 75% TWM, respectively; N100, NC with USM and UWM replaced with 100% TSM and 100% of TWM, respectively.

Values within a row without a common superscript differ significantly by LS means at *P* < 0.05.

## Discussion

Cellulase, β-glucanase, xylanases, β-mannanase, and pectinase have been shown to improve CP, energy, and mineral contents utilization in fibrous feedstuffs (Habte-Tsion and Kumar, 2018). The high AR of DM, NDF, and CP in pretreated WM and SM in the current study can be attributed to the effect of FDE on the solubilization of CP and fiber. This was achieved through the FDE`s ability to cleave β-linkages in NSP, releasing nutrients such as amino acids and sugars from the cell wall encapsulation (Kiarie et al., 2013; Yu et al., 2018). According to Yu et al. (2018), pretreatment of WM, wheat bran, corn, wheat, barley, and soybean meal with either 40,000 xylanase U/g or 5,000 β-glucanase U/g increased the AD of trace elements, xylan, and glucan when compared to a control. Given that lignin is resistant to even exogenous fiber-degrading enzyme hydrolysis (Knudsen, 2014), the lignin content (7.83%) of the SM used in the current study can explain the lower DM disappearance between its enzyme-pretreated and control samples.

Organic acids are released during the fermentation of oligosaccharides and monosaccharides, which affects pH (Broekaert et al., 2011). In general, grain soaked in water for a prolonged period will produce organic acids as a result of spontaneous fermentation facilitated by endogenous microflora and enzymes (Canibe and Jensen, 2012). The pH of the materials that were enzymatically pretreated in the current study showed a sharp decline in comparison to the control, indicating that endogenous microorganisms were fermenting carbohydrates into organic acids. In agreement with the current study, Rho et al. (2020) did not observe treatment and time interaction effects when WM was pretreated with fiber-degrading enzymes over time. However, there was an interaction between treatment and time on pH in SM in the current study, with the control having the highest pH at 24 h. Because SM is more lignified, endogenous microorganisms may have required greater than 24 h to hydrolyze the cell wall structure and utilize monosaccharides. Furthermore, since the plastic containers were tightly sealed throughout the incubation period, pretreatment in the current study was carried out in a semi-anaerobic environment. As a result, some of the released MS were fermented into organic acids (Broekaert et al., 2011). The total organic acid concentration peak in the WM, SM controls, and enzyme treatments occurred at 48 h.

The energy values of any grain and its byproducts may be affected by the makeup of their NSP (Jaworski et al., 2015). The main NSP in most cereal grains are β-glucan, arabinoxylan, and insoluble cellulose (Marcotuli et al., 2020). Because most feeds contain complex NSP, using multi-enzyme cocktails ensures that the enzymes work synergistically when hydrolyzing them (Ravindran, 2013; Kiarie et al., 2016). To target the various NSP found in each ingredient, the current study combined xylanase, cellulase, and β-glucanase for WM and a mixture of cellulase, β-glucanase, and β-mannanase for SM. This combination increased the amounts of xylose, arabinose, and glucose in the pretreated materials when compared to the corresponding controls. The observed increase in MS concentrations suggested that the NSP present in both WM and SM was successfully hydrolyzed by the FDE mixtures used in the current study. The concentration of MS in pretreated SM is consistent with findings made by Malathi and Devegowda (2001), who pretreated SM with 900 U/g xylanase and 12 FPU/g cellulase from *Trichoderma viridae* and observed an increase in total MS release when compared to the control without enzymes.

The concentration of MS in pretreated WM had no time or treatment interaction effect in the current study, indicating that there is no benefit to pretreating WM beyond 24 h, which is consistent with Rho et al. (2020) study in which WM was pretreated with 62,000 xylanase, 37,000 cellulase, and 8,000 β-glucanase U/g. After carbohydrates are depleted during fermentation, there is a shift to fermenting nitrogen sources like amino acids. This leads to an increase in the production of isobutyric acid (Jha et al., 2019). The current study found that WM pretreatment increased isobutyric production over time, with a peak at 48 h. To avoid nitrogen fermentation, the pretreatment of WM should not last more than 24 h. The lack of a treatment and time interaction effect on butyric acid concentration in the current study can be attributed to the rapid drop in pH. This drop in pH prevented the conversion of lactate to lactic acid, which may have then been converted to either butyric acid or acetic acid (Twomey et al., 2003; Bourriaud et al., 2005). When combined with fiber-degrading enzymes, proteases can increase protein solubilization. For instance, in a study where xylanase and protease were combined, protease increased CP solubilization during enzymatic pretreatment of corn-dried distillers’ grains with solubles (**DDGS**) and wheat DDGS (Pedersen et al., 2015). In the current study, adding protease to FDE increased CP solubilization in SM at 24 and 48 h. The proteases increased protein solubilization by breaking down insoluble protein aggregates or complexes into smaller, soluble fragments. By breaking down these larger protein structures, proteases can increase the amount of soluble protein in a sample (Muchiri et al., 2023).

 Increasing fiber intake enhances the muscularity and size of gizzard, improving its grinding actions of any ingested digesta (Jiménez-Moreno et al., 2019). Including moderate (2.5%) fiber sources in broiler chicken diets may promote the retention of components such as CP through promotion of reflux and digesta mixing from the proventriculus into the gizzard (Jiménez-Moreno et al., 2019; Sanchez et al., 2021; Njeri et al., 2023). However, if the level of dietary fiber is much higher, it will have a negative impact on the AR of components. It has been demonstrated that dietary fiber, particularly insoluble fiber, can speed up digesta transit time while decreasing digestibility (le Goff et al., 2002; Shi and Noblet, 1993). For instance, the digesta retention was decreased by 9 h in pigs fed a diet with wheat bran (207.4 g/kg total diet NDF) as a source of insoluble fiber (Wilfart et al., 2007). In the current study, the NC, which had 11.8% NDF (day 21) retained 7.53% more CP and 1.42% more AMEn than PC indicating that the NDF level had a positive effect on the birds. Further increment of NDF to 14.9% (day 42) decreased the energy retention but still promoted AR of CP by 1.06% relative to PC. The pig study by Wilfart et al. (2007) may help to explain why NC birds retained less DM, and energy on day 42 relative to PC birds. These findings support those of Dunaway and Adedokun (2019), who demonstrated that, compared to a corn-and-soybean-based diet, WM decreased energy and DM retention. Fiber-degrading enzymes like xylanase have been shown to increase fiber digestibility (Moran et al., 2016). Replacement of untreated SM and WM in the NC with pretreated SM and WM increased AMEn concentration and had a linear (day 21) and a quadratic (day 42) increment response. This AMEn increase can be attributed to enzymatic pretreatment solubilizing NSP of which the birds were able to utilize, thus increasing the AR of components such as energy and fiber. The linear increase in AMEn concentration can also be attributed to increased oil levels in the diet because of increased SM inclusion.

It is also important to note that the AR of CP and NDF in the N25, N50, N75, and N100 had a linear or a quadratic response. However, the retention of these components was reduced at higher inclusion levels of the pretreated materials (N75 and N100) than at lower levels (N25 and N50), suggesting that the FDE-pretreated materials affected the AR of the components at the higher inclusion levels. These findings can be attributed to how the diets were formulated, as the nutrients that FDE may have liberated were not taken into account. This led to an underestimation of the digestible/available nutrients in the pretreated materials. As a result, additional nutrients like amino acids that FDE may have released might have been excreted. Another contributing factor could be the concentration of xylose and arabinose in the pretreated materials. When compared to the control, the xylose concentrations in pretreated WM and SM were 59.44 and 29.40 mol/mL, respectively, with pretreated WM also containing 4.01 mol/mL arabinose. If these two MS (xylose and arabinose) were absorbed at a higher concentration, there is a possibility that they caused adverse metabolic effects, even though their concentrations in the blood were not measured in the current study (Regassa et al., 2017). The effect of xylose and arabinose on metabolism may also explain some of responses seen in the current study and companion growth performance as reported by (Muchiri et al., 2023). Specifically, pretreated feedstuffs increased liver weight linearly, with birds fed N100 having the heaviest liver. This indicated that the pretreated feedstuffs may have had a direct negative effect on the liver. Inclusion of more than 5% level of xylose in broiler chicken diets damaged the liver by depressing the expression of enzymes involved in lipid and glucose metabolism. This may keep the body in a catabolic state to release gluconeogenic precursors, thus promoting hepatic hypertrophy (Regassa et al., 2017; Muchiri et al., 2023).

The moisture content of excreta can be used to assess litter quality (Kimiaeitalab et al., 2017). van der Hoeven-Hangoor et al. (2014) observed that adding a source of insoluble fiber (coarse oat hull) to a wheat-based broiler chicken diet reduced excreta moisture when compared to a corn-based control diet. However, their study was on floor pens with wood shavings, which could have contributed a significant source of insoluble fiber through ingestion of bedding materials. In the current study, untreated SM and WM reduced excreta moisture when compared to the control. The substitution of untreated with pretreated SM and WM reduced moisture linearly (day 21) or quadratically (day 42). This indicated that untreated SM and WM may have contributed a significant amount of insoluble fiber leading to the moisture reduction.

Broiler chickens are thought to develop a compensatory mechanism by increasing their mucosal surface to improve nutrient absorption when they are fed diets low in energy (Bedford, 2000). In a study conducted by Röhe et al. (2020), it was shown that the concentrations of cecal acetic and propionic acids were reduced when dual-purpose laying hens were fed a high-fiber and low energy diet in comparison to a control. In addition, the dual-purpose laying hens had an increased intestinal mucosal surface relative to the controls as a compensatory mechanism for the reduced energy in the diet. In the current study, NC, N25, N50, N75, and N100 total cecal sugars and organic acids were lower than PC. This may be because the NSP in the test diets was hydrolyzed to MS, which could have been directly absorbed in the small intestines, limiting the available fermentable substrate at the cecal level. Furthermore, although the current study did not measure mucosal surface, based on Röhe et al. (2020), it can be hypothesized that the birds also developed compensatory mechanisms for energy deficiency by enhancing absorption of cecal fermentation precursors (glucose, xylose, arabinose, etc.). The birds might have adapted to the lower energy diets by increasing their mucosal surface (NC and test finisher diets were reduced by 150 kcal/kg relative to PC). Further supporting the hypothesis, In the current study, total MS concentrations changed between days 21 and 42 diets. The bird maturation and adaptation to a high NSP diet can be linked to an increase in the mucosal surface to enhance MS absorption.

In comparison to the corresponding controls, pretreating of WM and SM with FDE increased the solubilization of CP and the coefficients of AD of fiber. The FDE also elevated the concentrations of organic acids and MS. Incubating sunflower with FDE and protease increased CP solubilization. The addition of FDE-pretreated SM and WM to a corn–soybean meal diet for broiler chickens at N25 and N50 levels resulted in an increase in nutrient and energy retention. However, it is crucial to conduct further research to identify any potential harmful factors associated with the inclusion of high levels of pretreated feedstuffs (N75 and N100).
